# Simultaneous EGFR and VEGF Alterations in Non-Small Cell Lung Carcinoma Based on Tissue Microarrays

**Published:** 2007-01-12

**Authors:** Evangelos Tsiambas, Athanasios Stamatelopoulos, Andreas Karameris, Ioannis Panagiotou, Dimitrios Rigopoulos, Antonios Chatzimichalis, Demosthenes Bouros, Efstratios Patsouris

**Affiliations:** 1 Department of Pathology, Tissue Microarrays and Computerized Image Analysis Laboratory, 417 VA Hospital (NIMTS), Athens, Greece; 2 Department of Thoracic Surgery, “Metaxa” Hospital, Pireaus, Greece; 3 Departmentt of Pathology, Medical School, University of Athens, Greece; 4 Department of Thoracic Surgery, 401 GA Hospital, Athens, Greece; 5 Department of Internal Medicine, 401 GA Hospital, Athens, Greece; 6 Department of Respiratory Diseases, “Demokrition” University of Thrace, Alexandropole, Greece

**Keywords:** Non-small cell lung carcinoma, tissue microarrays, genes, epidermal growth factor receptor, vascular endothelial growth factor

## Abstract

**Background:**

Epidermal growth factor receptor (EGFR) overexpression is observed in significant proportions of non-small cell lung carcinomas (NSCLC). Furthermore, overactivation of vascular endothelial growth factor (VEGF) leads to increased angiogenesis implicated as an important factor in vascularization of those tumors.

**Patients and Methods:**

Using tissue microarray technology, forty-paraffin (*n* = 40) embedded, histologically confirmed primary NSCLCs were cored and re-embedded into a recipient block. Immunohistochemistry was performed for the determination of EGFR and VEGF protein levels which were evaluated by the performance of computerized image analysis. EGFR gene amplification was studied by chromogenic *in situ* hybridization based on the use of EGFR gene and chromosome 7 centromeric probes.

**Results:**

EGFR overexpression was observed in 23/40 (57.5%) cases and was correlated to the stage of the tumors (*p* = 0.001), whereas VEGF was overexpressed in 35/40 (87.5%) cases and was correlated to the stage of the tumors (*p* = 0.005) and to the smoking history of the patients (*p* = 0.016). Statistical significance was assessed comparing the protein levels of EGFR and VEGF (*p* = 0.043, *k* = 0.846). EGFR gene amplification was identified in 2/40 (5%) cases demonstrating no association to its overall protein levels (*p* = 0.241), whereas chromosome 7 aneuploidy was detected in 7/40 (17.5%) cases correlating to smoking history of the patients (*p* = 0.013).

**Conclusions:**

A significant subset of NSCLC is characterized by EGFR and VEGF simultaneous overexpression and maybe this is the eligible target group for the application of combined anti-EGFR/VEGF targeted therapies at the basis of genetic deregulation (especially gene amplification for EGFR).

## Introduction

Although the design and development of novel therapeutic strategies demonstrate encouraging results in patients who suffer from non-small cell lung carcinoma (NSCLC), there is an increased need for a more rational selection of those patients who could earn survival benefits by the combination of conventional, systemic chemotherapy and targeted therapy, such as monoclonal antibodies or intracellular tyrosine-kinase inhibitors (Baselga and Arteaga, 2006). Disruption of crucial signal transduction pathways, which under molecular deregulation lead to upregulated proliferation, metastasis, and angiogenesis, is a challenge for combined targeted therapeutic strategies (Liebmann, 2001).

Epidermal growth factor receptor (EGFR) gene is located on chromosome 7 (7p12) and its product is a 170 kDa protein, comprising of three major functional domains: an extracellular ligand-binding domain, a hydrophobic transmembrane domain and a cytoplasmic tyrosine-kinase domain (Harari, 2004). Ligands, such as EGF or TGF-α, bind to the extracellular domain of the receptor and trigger a cataract of reactions, including dimerization and phosphorylation of the intracellular domain and finally signal transduction to nucleus is mediated by the involvement of RAS/RAF/MAPK proteins predominantly and via an alternative pathway (PI3/AKT/mTOR) (Molina and Adjei, 2006). In aggressive tumors, such as glioblastomas, some studies have shown that EGFR gene amplification is correlated to shorter survival time and resistance to radiotherapy (Marquez et al. 2004). Almost recently, novel targeted therapeutic strategies including anti-EGFR agents, such as monoclonal antibodies (cetuximab) and small molecules (gefitinib, erlotinib) have been approved for the treatment in some types of EGFR-dependent cancers, such as colon or lung and pancreatic (Barder et al. 2004; Jensen et al. 2006; Krempien et al. 2005). Although EGFR protein overexpression is observed in significant proportions (~40 to 80%) of NSCLCs, the crucial process for a successive targeted therapeutic approach (survival benefits) remains the identification of specific gene deregulation mechanisms (Ji et al. 2006; Kosaka et al. 2004). Some studies have already shown the association between specific mutations of EGFR tyrosine-kinase intracellular chains and the response to small molecules acting as inhibitors in this domain, such as gefitinib or erlotinib (Mukohara et al. 2005; Perez-Soler et al. 2004; Kris et al. 2003). In contrast, there are controversial results regarding to the efficacy of monoclonal antibodies therapy in those patients (Raben et al. 2005; Thienelt et al. 2005). Furthermore, EGFR gene amplification seems to be a relatively rare (∼5% to 15%) but significant molecular deregulation mechanism affecting the response to those agents (Hirch et al. 2003).

Vascular epithelial growth factor (VEGF), also, has been characterized as the key mediator of angiogenesis in cancers of different types (Ellis et al. 2006). VEGF gene is a member of the PDGF/VEGF growth factor family and is located on chromosome 6 (6p12). Its protein product (isomorph A) is a glycosylated mitogen acting as an endothelial cell growth factor, promoter of cell migration, and inhibitor of apoptosis (Voelkel et al. 2006). Although this cytokine normally induces endothelial proliferation and increases vascular permeability, it is also involved in tumor-associated angiogenesis by its overexpression (Zhu et al. 2006). Under hypoxic conditions, HIF 1α-α transcription factor responsible for the regulation of oxygen homeostasis—is activated through PI3 kinase–AKT and MAPK–ERK pathways, binding with its complementary factor HIF 1β to the promoters of genes that mediate glycolysis and angiogenesis, such as VEGF (Chang et al. 2006; Strieter, 2005). Aberrant secretion of VEGF due to hypoxia, activation of oncogenes, and even EGFR or an abnormal hormonal activity leads to an uncontrolled binding to specific receptors such as VEGFR-1or VEGFR-2 (Langer and Natale, 2005; Matsumori et al. 2006). This process triggers a cataract of reactions including phosphorylation of intracellular tyrosine-kinase chains and finally leads to tumor angiogenesis characterized by an abnormal structurally and functionally vasculature (Thorpe, 2004; Siemann and Shi, 2004). Concerning NSCLC, some recently published Phase II–III studies have shown encouraging results due to combination of anti-VEGF monoclonal antibodies (bevacizumab) and chemotherapeutic agents, such as carboplatin, paclitaxel or anti-EGFR inhibitors, such as cetuximab or gefitinib (Morelli et al. 2006; Robert et al. 2005; Hebst et al. 2005). Despite this progress in the management of those patients, identification of specific molecular criteria is a challenge for increased response rates (Pao et al. 2004).

Using tissue microarrays (TMAs) and computerized image analysis (CIA) we evaluated EGFR and VEGF protein expression by Immunohistochemistry (IHC) and also we analyzed EGFR gene status by Chromogenic *in situ* Hybridization (CISH) in order to identify potential significant correlation of these two genes in NSCLCs.

## Materials and Methods

We obtained, for the purposes of our study, forty (*n* = 40) formalin fixed and paraffin embedded archival tissue samples of histologically proven NSCLC including 27 adenocarcinomas (AC), 2 bronchioloalveolar carcinomas (BAC), 9 squamous cell carcinomas (SCC) and 2 large cell carcinomas (LCC). Most of them were initially diagnosed by the performance of CT guided fine needle aspiration (FNA), using ThinPrep method (Cytyc, U.S.A.). According to our therapeutic protocols, the patients classified as stage I and II received only surgical therapy (radical ablation: lobectomies and pneumonectomies, associated with radical lynphadenectomy). Patients in stage IIIa or IIIb had been projected to follow new adjuvant chemotherapy (*cis*-platin, vinorelbine, gemcitabine or combined treatment between them), radiotherapy (2000–6500 cGy) and in case of shrink of the initial mass, 2–3 weeks after the therapy were submitted for surgical ablation of the tumor. Surgical specimens were obtained by the performance of open minimal surgery biopsies (VATS), in some cases of stage III and the only one IV case included in this study. All corresponding Hematoxylin and Eosin (H&E)-stained slides were reviewed by two pathologists for confirmation of diagnosis and classification according to World Health Organization (WHO) grading and staging (TNM system) criteria for lung cancer. The tissue samples were referred to 31 male (mean age: 57) and 9 female (mean age: 62) patients. Clinicopathological data are demonstrated in Table 1.

### TMA construction

Areas of interest were identified in H&E stained slides by a conventional microscope (Olympus BX-50, Melville, NY, U.S.A.). Selection of those areas was performed on the basis of tumor sufficiency and accurate histological confirmation, avoiding sites of necrosis or bleeding. The corresponding paraffin blocks were obtained for the construction of one TMA block. Using ATA-100 apparatus (Chemicon International, Temecula, CA, U.S.A.), all of the source blocks were cored two times (in order to secure the presence of each case in the final block) and 1-mm diameter tissue cylindrical cores were transferred from each conventional donor block to the recipient block. The final constructed TMA block contained 85 cores of tissue cylindrical specimens, including five cores of the control group (normal appearing lung epithelia). After 3 mm microtome sectioning and H&E staining, we observed microscopically that all examined cases were represented by at least one (7 cases) or two tissue spot (33 cases)-confirmation of the adequacy of cylindrical specimens.

### Antibodies and probes

Ready-to-use EGFR monoclonal mouse antibody (clone 31G7-Zymed/InVitrogen, San Fransisco, U.S.A.) recognizing predominantly the extracellular domain of EGFR protein and not reacting with other erbB receptors used. Similarly, anti-VEGF polyclonal antibody (LYL-Biogenex, San Ramon, CA, U.S.A.) recognizing the Aisoform applied. EGFR gene status was determined using the ready to use SPOT LIGHT EGFR DNA Probe (Zymed/InVitrogen, San Fransisco, U.S.A.). This digoxygenin-labeled probe is located on 7p12 and covers the entire EGFR gene area. A chromosome 7 status was determined by the ready to use biotin-labeled chromosome 7 centromeric probe (Zymed/InVitrogen, San Fransisco, U.S.A.) recognizing the specific repetitive centromeric DNA sequences known as α-satellite DNA.

### Immunohistochemistry (IHC)

**Figure 1.**
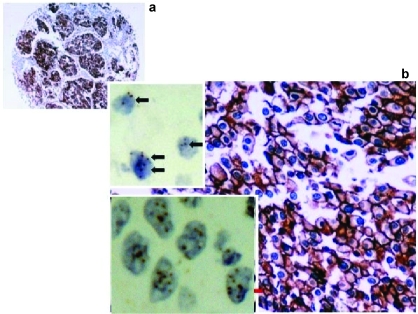
EGFR overexpression was found in a case of NSCLC. (**A**) A tissue microarray core (diameter 1 mm) demonstrating EGFR high value of protein expression (conventional score 3+). Original magnification: 10× (**B)**. Note in the same case the specific—for protein overexpression-complete, “ring like”, dense membranous predominantly immunostaining pattern (clone 31G7). Original magnification: 40× **upper inside.** Chromosome 7 aneuploidy by CISH analysis. Note 3 and 4 centromeric signals per nucleus (black arrows) **down inside**. EGFR gene amplification by CISH analysis. Note 6–10 gene copies as dark blue scattered signals or small clusters of them (red arrows) per nucleus. Original magnification for CISH: 40×.

As described above in the paraffin sections of TMA block, the IHC for EGFR and VEGF antigens was carried out on 3 μm serial. Two slides were deparaffinized and rehydrated. The first of them was enzyme digested (proteinase K) for 10 min at 37 °C. The NBA kit (Zymed/InVitrogen, San Fransisco, U.S.A) was used for the following detection steps. Blocking solution was applied to the slides for 10 min, followed by incubation for 1 h using the EGFR monoclonal antibody (dilution 1:10) at room temperature. Following incubation with the secondary antibody for 10 min, diaminobenzidine-tetrahydrocloride-DAB (0.03%) containing 0.1% hydrogen peroxide was applied as a chromogen and incubated for 5 min. Sections were counterstained, dehydrated and cover-slipped. For VEGF expression, the second slide was incubated for 1 h using the VEGF polyclonal antibody (dilution 1:60) without enzyme digestion. For negative control slides, the primary antibodies were omitted. Both IHC protocols were performed by the use of an automated staining system (I 6000–Biogenex, San Ramon, CA, U.S.A.). Membranous and submembranous cytoplasmic staining was considered acceptable for those two markers, respectively, according to manufacturer’s data sheet. (Fig. 1a). Colon cancer tissue sections overexpressing EGFR and VEGF, and normal appearing colon and lung epithelia were used as a positive and negative control, respectively. EGFR protein expression levels were evaluated semi-quantitatively by using Zymed’s Evaluation Guidelines. According to the scoring guidelines, the examined cases were classified as follows: Score 0: no staining or membrane staining in <10% of tumor cells; Score 1+: faint membrane staining in >10% of tumor cells; Score 2+: weak or moderate complete membrane staining in >10% of tumor cells and Score 3+: strong, complete membrane staining in >10% of tumor cells. Scores of 0 and 1+ were considered as negative for EGFR expression while Scores 2 + and 3 + as positive (overexpression). VEGF protein levels were evaluated semi-quantitatively also using the modification described above predominantly for cytoplasmic/membranous focal or diffuse staining pattern.

### Chromogenic in situ hybridization (CISH)

CISH SPOT-Light Chromogenic ISH Detection Kit was applied. CISH for chromosome 7 status and EGFR gene analysis was performed on 5 μm thick paraffin serial sections of the TMA block described above. Two slides were incubated at 37 °C overnight followed by 2 h incubation at 60 °C and then deparaffinized in xylene two times, 5 min each and in ethanol three times, 3 min each. The slides were rinsed in deionised water and then placed in a coplin jar containing CISH FFPE Pretreatment Buffer (CISH Tissue Pre-treatment Kit, Zymed). For heat pre-treatment, the coplin jar was capped, loosely screwed, placed in a pressure cooker and timed for 10 min after the pressure built up. The slides, then, were immediately washed in deionised water followed by enzyme digestion, which was performed by covering the sections with pepsin (CISH Tissue Pre-treatment Kit, Zymed) for 5 min at 37 °C. The slides were washed with deionised water, dehydrated with graded ethanol and air-dried. Ready to use dig-labeled EGFR gene and biotin-labeled chromosome 7 centromere probe was applied to each section, respectively. Twenty microliter of probe was applied to each of the TMA sections. The tissue sections containing the added probe were denatured by placing the slides in a polymerase chain reaction (PCR) machine equipped with a slide block at 94 °C for 5 min. The slides were then placed in a moist slide box and incubated at 37 °C for overnight hybridization. The sections were stringently washed in 0.5x standard saline citrate at 75 °C for 5 min. The CISH Polymer and the Horseradish (HRP) Detection Kit (Zymed/InVitrogen, San Fransisco, U.S.A.)—containing similar steps to IHC—were used. Shortly, afterwards TMA sections were placed in 3% H_2_O_2_ and diluted with methanol for 10 min to block endogenous peroxidase. To block unspecific staining, Cas Block^TM^ (Zymed/InVitrogen, San Fransisco, U.S.A.) was applied and incubated for 10 min. Following incubation with mouse anti-dig for 30 min and then polymerised HRP conjugated anti-mouse for 30 min, the EGFR probe was visualized by DAB development (CISH Polymer Detection Kit, Zymed). The biotin labeled Chr 7 centromere probe was detected by incubation with HRP conjugated streptavidin for 30 min, followed by DAB development (CISH Centromere Detection Kit, Zymed) for 30 min. TMA sections were lightly counterstained with hematoxylin and dehydrated in graded ethanol. At the end of the process, CISH centromere signals or gene copies were easily visualized as dark brown/blue scattered or in small clusters dots, using a conventional, bright-field microscope (Fig. 1b).

Interpretation of EGFR gene and chromosome 7 centromere signal results was based on Zymed’s Evaluation Chart for CISH. According to this guide, two gene copies per nucleus demonstrate normal EGFR gene pattern, whereas 6–10 or small clusters characterize a low-level gene amplification. In this case, chromosome 7 status must be evaluated to exclude aneuploidy (3–5 centromeric signals per nucleus; diploid pattern demonstrates normal chromosome status). Finally, high gene amplification level is characterized by the presence of more than 10 gene copies or large clusters of them per nucleus in more than 50% of the examined cells.

### Computerized image analysis (CIA)

**Figure 2.**
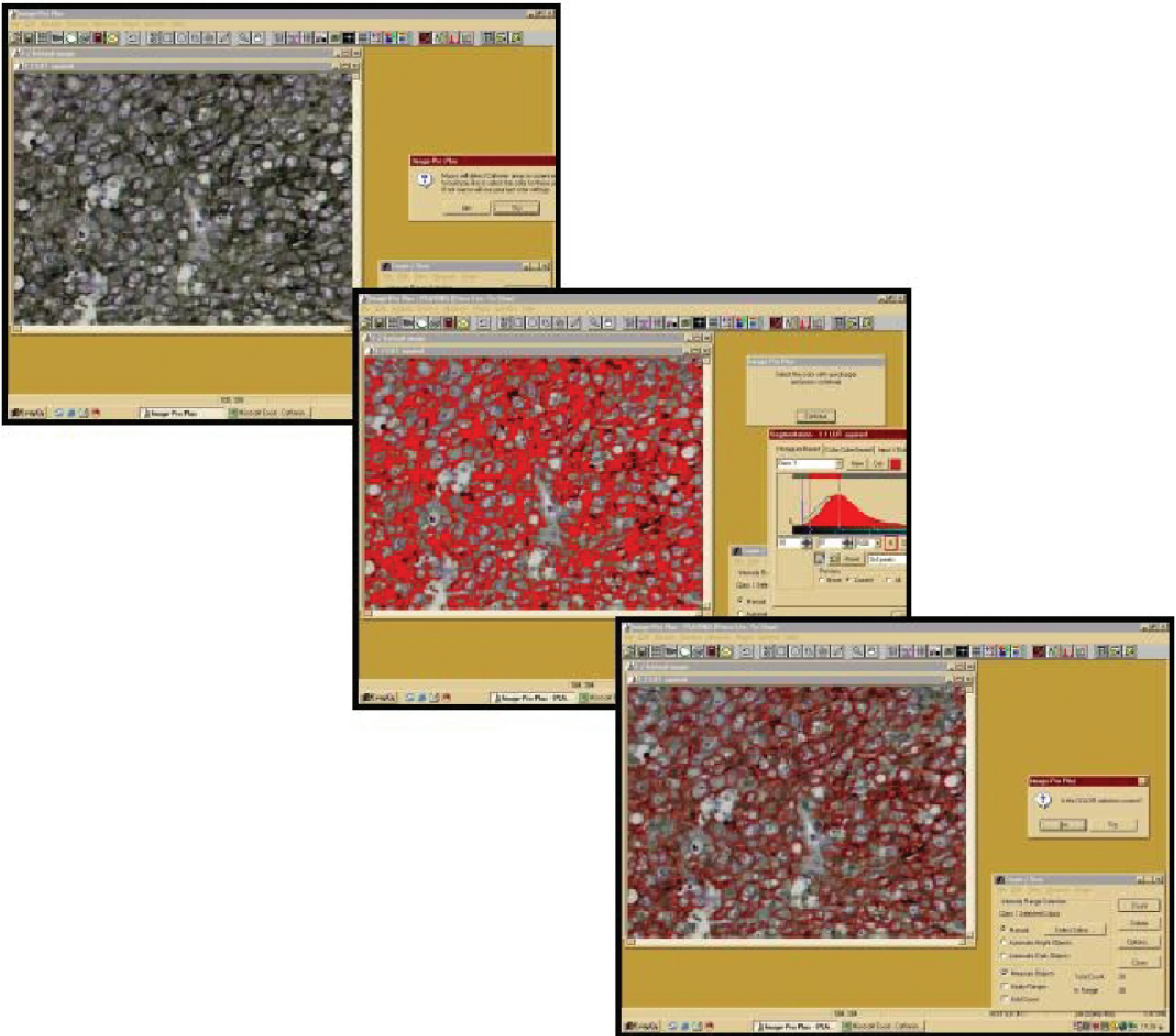
VEGF protein expression evaluated by Computerized Image analysis shows Reddish areas that represent cytoplasmic and membranous immunostaining.

In order to evaluate the IHC results-specifically 2+ and 3+, we semi-quantitatively characterized cases-in an accurate and faster way. Then, we perform CIA by using a semi-automated system with the following hardware features: Intel Pentium IV, MATROX II Card Frame Grabber, Digital Camera Microwave systems (800 × 600), Microscope Olympus BX–50 and the following software: Windows XP/Image Pro Plus, version 3.0-Media Cybernetics 1997. Measurements of EGFR and VEGF staining intensity values were performed in five optical fields per case and at a magnification of 400 × (Fig. 2). Using normal epithelia as control group and basis for the evaluation of protein expression levels, we compared them to the examined tumors. We observed that EGFR and VEGF expression levels—characterized as Low, Moderate and High-ranged between discrete values. Interpretation of staining intensity values (range 0–255) is demonstrated in Table 2.

### Statistical analysis

Associations between variables including protein expression levels, gene, and chromosome status and clinicopathological parameters such as gender, tumor histology, smoking history, grade and stage were performed by the application of χ test estimated along with its 95% CI (SPSS Inc., Chicago IL v.11.0). Two tailed *p* values < 0.05 were considered statistically significant. Cohen’s inter-rater kappa was also estimated along with its 95% CI to evaluate concordances between the two examined proteins. By its definition, a κ value of 1 denotes complete agreement, values of more than 0.75 are characterized as excellent agreement, values between 0.40 and 0.75 show fair to good agreement, values more than 0 but less than 0.40 show poor agreement, and a kappa value of 0 indicates that the observed agreement is equal to chance. Total (IHC and CISH) results are described in Table 2.

## Results and Analysis

### EGFR and VEGF IHC assessment

IHC results were successfully obtained from all the forty NSCLC cases. EGFR overexpression was observed in 23/40 (57.5%) cases. Concerning histological type, protein overexpression was observed in 12/27 ACs, 8/9 SCCs, 1/2 BACs and 2/2 LCCs. According to the conventional evaluation criteria, 8 cases were evaluated as 2+ and 15 cases as 3+. Computerized image analysis for EGFR protein staining intensity levels showed that 6 cases demonstrated moderate values, whereas 17 cases high values. EGFR protein expression was statistically associated with stage (*p* = 0.001), but not with grade (*p* = 0.325), and histological type of the examined tumors (*p* = 0.133). Specifically, biphasic EGFR immunostaining pattern (membranous and cytoplasmic) was observed in 12/23 (52.1%) cases and interestingly was found to be correlated to advance stage (*p* = 0.001), and also to grade (*p* = 0.046). Additionally, 1/5 cases of the normal appearing epithelia demonstrated moderate value of EGFR protein overexpression (2+). EGFR protein levels were not associated to gene status (*p* = 0.241), chromosome status (*p* = 0.489), and smoking history (*p* = 0.733), respectively.

VEGF overexpression was observed in 35/40 (87.5%) cases. Concerning histological type, protein overexpression was observed in 23/27 ACs, 9/9 SCCs, 2/2 BACs and 1/2 LCCs. According to the conventional evaluation criteria, 17 cases were evaluated as 2+ and 18 cases as 3+. Computerized image analysis for VEGF protein staining intensity levels showed that 20 cases demonstrated moderate values, whereas 15 cases high values. VEGF protein expression was statistically associated with stage (*p* = 0.005) and smoking status (*p* = 0.016), but not with grade (*p* = 0.229), and histological type of the examined tumors (*p* = 0.211). All those protein overexpression cases demonstrated a biphasic immunostain pattern (membranous and diffuse cytoplasmic). Additionally, 2/5 cases of the normal appearing epithelia demonstrated moderate value of VEGF protein overexpression (2+). Interestingly, by correlating EGFR to VEGF protein levels, we observed a statistical significance (*p* = 0.043), and a high interrater kappa value (*k* = 0.846). Combined EGFR and VEGF overexpression (overall High and Moderate values) was observed in 22/40 (55%) cases and a statistical significance was established correlating to stage of those tumors (*p* = 0.001).

### EGFR gene and chromosome 7 CISH assessment

CISH results were also successfully obtained from all the examined cases. In cases with EGFR gene amplification, 6–10 scattered copies were detected in cell sub-populations (low amplification status). This genetic event was observed in 2/40 cases (5%) linked to histological type (both of them were AC cases) and to normal (diploid) chromosome status. In EGFR gene, numerical alterations (amplification) were not associated with the parameters already referred (grade: *p* = 0.276, stage: *p* = 0.330, smoking history (*p* = 0.076) and histological type: *p* = 0.798). Chromosome 7 instability (aneuploidy, observed as 3–5 dots per nucleus) was detected in 7/40 (17.5%) cases. All other cases were diploid with regard to chromosome 7 status (2 signals per nucleus). Significant statistical correlation was not observed by correlating chromosomal status to those parameters as they are mentioned before (*p* = 0.757, *p* = 0.297, and *p* = 0.540, respectively) and also to EGFR gene status (*p* = 0.504). Interestingly, by correlating chromosome 7 pattern to smoking history of the patients, we observed a strong statistical significance (*p* = 0.013). Finally, we did not detect gene or chromosome numerical alterations in the control group (normal epithelia).

## Discussion

Our study was designed to investigate the association between EGFR and VEGF alterations in NSCLCs based on a TMAs substrate. Although a significant proportion of the examined tumors demonstrated EGFR protein overexpression, the specific mechanism of gene amplification was identified only in a small percentage (5%). The highest rate of EGFR strong positivity (3+/High staining intensity values) was observed in SCCs (88.8%). In contrast, gene amplification was detected by CISH in two cases of ACs overexpressing EGFR. Other studies using PCR, FISH or CISH method have shown different proportions of EGFR gene amplification and there are controversial results correlating protein expression and gene status (Cappuzo et al. 2006; Awaya et al. 2005; Reinmuth et al. 2000). It seems that the majority of EGFR overexpressed NSCLCs, especially SCCs are associated to different molecular deregulation mechanisms than amplification regarding EGFR gene (Ekstrand et al. 1992). Short deletions and point mutations have been already identified in NSCLC patients (Janne et al. 2006). Exons 18–21 that encode the intracellular ATP-binding domain (tyrosine-kinase chains) represent “hot spots” of somatic mutations, including deletions in exon 19, small in-frame insertions in exon 20 and mis-sense mutations in exons 18–20 (Yang et al. 2006). Large deletions of the extracellular domain of the receptor are rare-genetic events in NSCLC comparing to other neoplasms, such as glioblastomas (Moscatello et al. 1995; Frederick et al. 2002). Another mechanism of EGFR overactivation is the establishment of an autocrine feedback loop. Self-production of EGF or TGF-α ligands leads to receptor activation and finally enhanced signaling. A study showed that among EGFR-positive primary lung ACs, overall survival was significantly poorer for patients demonstrating high protein levels of those ligands than the others, which characterized by low or negative expression (Tateishi et al. 1990). Almost recently, another study analyzing EGFR gene status by FISH and PCR methods identified a subset of patients who were characterized by simultaneous gene amplification and point mutations (Argiris et al. 2006).

Prognostic implication of EGFR protein expression levels, somatic mutations or/and gene copy number remains under investigation. Some studies have shown controversial results with regard to the response rate in agents, such as monoclonal antibodies or intracellular inhibitors. Among them statistical significance has been achieved associating EGFR expression and/or point mutations to monotherapy or combined therapy strategies including gefitinib, erlotibin and conventional chemotherapeutic drugs (Giaccone et al. 2004; Pao et al. 2006; Han et al. 2006). In contrast, application of monoclonal antibody targeted therapies is under investigation. Phase II–III studies using cetuximab, a monoclonal human-murine chimeric antibody against the extracellular domain of the receptor, showed that a small subset of patients might earn survival benefits (Baselga et al. 2000; Rosell et al. 2004). Using colon cancer tissues as a substrate for combined IHC and molecular analysis (FISH and PCR), a recently published study showed that EGFR copy number and non-protein overexpression is linked with prognosis and patients demonstrated this specific gene deregulation mechanism probably earn survival benefits, responding to monoclonal antibody treatment (Moroni et al. 2005). In our study, we used a monoclonal antibody targeting predominantly the extracellular domain of the receptor for IHC analysis, because cetuximab binds to this site.

In the current study, also, VEGF protein expression levels were associated to stage of the examined tumors and to smoking status. Cigarette smoke is responsible for the multiple genetic abnormalities observed in lung cancer, even before morphologic changes are identified in the normal appearing bronchial mucosa by conventional light microscopy (Sekido et al. 2003). In our study, we found a statistical significance correlating smoking history and chromosome 7 instability, reflecting probably this genetic imbalance. Interestingly, a high concordance was assessed analyzing EGFR and VEGF co-expression. Both of those proteins are involved in the angiogenetic process and for this reason cetuximab and bevacizumab—a recombinant humanized anti-VEGF monoclonal antibody—share similar activity (Ciardiello et al. 2000). A number of studies have proven a clear correlation between VEGF expression, microvessel density, and impaired prognosis. In addition, encouraging results regarding survival status were observed by the application of combined targeted (monoclonal antibodies) and conventional therapeutic regiments (Lyseng-Williamson et al. 2006; Gridelli et al. 2006). Furthermore, imbalances between receptors and their ligands, such as VEGFR-3 and VEGF-C are potentially involved in the progression of NSCLC promoting lymph node metastasis (Takizawa et al. 2006).

For quite a long time, IHC has been the method of protein expression evaluation in pathologic samples. Despite its relatively low cost and straightforward concept, immunostaining results can be divergent mainly due to varying sensitivity and specificity of commercially available antibodies, differences in tissue processing, lack of universal standard and inter observer differences in evaluating the staining results due to subjectiveness (Park et al. 2003). In the present study, co-evaluation of the EGFR gene status with EGFR protein overexpression provides greater insight and meaningful information than EGFR protein overexpression alone. Detection of molecular alterations, such as aneuploidy or gene deletion/amplification, improves the level of discrimination between sub-groups of patients and provides valuable molecular information for application of targeted therapies. Recently, CISH has drawn more attention, since it is capable of evaluating gene amplification/deletion, chromosome aneuploidy or chromosomal translocations simultaneously with tissue morphology on the same slide, using routine light microscopy under low magnification. Several studies have shown that CISH is an alternative to fluoresence *in situ* hybridization (FISH) method and both of them demonstrate a high level of concordance, comparing their results (92–98%) in the cases of HER2/neu gene status assessment (Park et al. 2003). It has been used to detect accurately and practically EGFR gene and Chr 7 status in pancreatic ductal adenocarcinomas or in glioblastomas and almost recently in NSCLCs (Tsiambas et al. 2006a,b; Koynova, 2005).

CIA methods can be applied to both Cytology and Histology (Gil 2002). Comparing the results obtained by the performance of conventional estimation (2+, 3+) to those of quantitative analysis (staining intensity values), we observed that although there is a strong concordance (100%) in the overexpression group, computerized image analysis is a more accurate method than the conventional eye microscopy for the evaluation of borderline protein expression levels. During digital analysis procedure cases characterized as borderline by conventional eye microscopy evaluation, (2+ or 3+) were found to be distinct (different numerical values). Cases characterized as High level staining intensity demonstrated values ranging between 67 and 112. Therefore, there was a ”window” of values (6 gray scale levels) comparing them to Moderate level of staining intensity (values ≥ 118), which was impossible to be calculated by conventional interpretation performed by the responsible pathologist. This observation is easily explained because the human eye can distinguish less than 200 gray levels of staining intensity. In contrast, commercial available image analysis software based on 8-bit or higher processors discriminate at least 256 continuous intensity values providing accurate results for the interpretation of immunohistochemical staining, according to our previous published experience (Tsiambas et al. 2006c).

In conclusion, we support the idea that EGFR and VEGF molecules play an important role in the progression and biological behavior of NSCLC (correlation to stage of the tumors). Identification of patients who are eligible for combined anti-EGFR/VEGF targeted therapeutic strategies has been based on EGFR gene deregulation mechanism (amplification or mutations) combined to VEGF overexpression.

## Figures and Tables

**Table 1.** Clinicopathological data (NSCLC cases).

		***n*= 40**	**%**
Gender	Male	31	78
	Female	9	22
Histology	AC	27	68
	SCC	9	22
	BAC/LCC	2/2	10
Grade	1	6	15
	2	18	45
	3	16	40
Stage	I	7	18
	II	16	40
	III/IV	17	42
Smoking status	Non	16	40
	Active	18	45
	Former	6	15

Ac, adenocarcinoma; SCC, squamous cell carcinoma; BAC, bronchioalveolar adenocarcinoma; LCC, large cell carcinoma. The Department of Pathology (417 VA Hospital-NIMTS, Athens, Greece) the local ethical committee gave permission to use those tissues for research purposes. Oral informed consent was obtained from each patient and the study protocol conforms to the ethical guidelines of the “World Medical Association Declaration of Helsinki—Ethical Principles for Medical Research Involving Human Subjects” adopted by the 18th WMA General Assembly, Helsinki, Finland, June 1964, as revised in Tokyo 2004.

**Table 2.** Combined IHC and CISH results.

***n*= 40**	**EGFR gene status**		***p value***	**Chr 7 status**		*p value*
	Normal	Amplification		Normal	Aneuploidy	
**EGFR IHC**			0.241			0.489
2+/3+ (M/H values)	21	2		18	5	
0/1+	17	0		15	2	
**VEGF IHC**			0.349			0.917
2+/3+ (M/H values)	33	2		29	6	
0/1+	5	0		4	1	

M/H values: Staining intensity values represent gray scale levels between 0 (black) and 255 (white). In this study, Low/Negative values ranged between 132–255, (0/1+), Moderate (M) values ranged between 118 and 131, whereas High (H) values ranged between 67–112

P values: chi square test (Cl 99%)

EGFR IHC vs VEGF IHC: Kappa (Cl 95%) = 0.846
